# Exploring the environmental diversity of kinetoplastid flagellates in the high-throughput DNA sequencing era

**DOI:** 10.1590/0074-02760150253

**Published:** 2015-12

**Authors:** Claudia Masini d’Avila-Levy, Carolina Boucinha, Alexei Kostygov, Helena Lúcia Carneiro Santos, Karina Alessandra Morelli, Anastasiia Grybchuk-Ieremenko, Linda Duval, Jan Votýpka, Vyacheslav Yurchenko, Philippe Grellier, Julius Lukeš

**Affiliations:** 1 Fundação Oswaldo Cruz, Instituto Oswaldo Cruz, Laboratório de Estudos Integrados em Protozoologia, Coleção de Protozoários, Rio de Janeiro, RJ, Brasil; 2 University of Ostrava, Life Science Research Centre, Ostrava, Czech Republic; 3 Russian Academy of Sciences, Zoological Institute, Laboratory of Molecular Systematics, St Petersburg, Russia; 4 Universidade do Estado do Rio de Janeiro, Instituto de Biologia Roberto Alcântara Gomes, Departamento de Ecologia, Rio de Janeiro, RJ, Brasil; 5 Sorbonne Universités, Muséum National d’Histoire Naturelle, Centre National de la Recherche Scientifique, Unité Molécules de Communication et Adaptation des Microorganisme, Unités Mixte de Recherche 7245, Paris, France; 6 Czech Academy of Sciences, Institute of Parasitology, Biology Centre, České Budejovice, Czech Republic; 7 Charles University, Faculty of Science, Department of Parasitology, Prague, Czech Republic; 8 University of South Bohemia, Faculty of Sciences, České Budejovice, Czech Republic; 9 Canadian Institute for Advanced Research, Toronto, Canada

**Keywords:** Kinetoplastea, metagenomics, metabarcoding, taxonomy, Trypanosomatida

## Abstract

The class Kinetoplastea encompasses both free-living and parasitic species from a wide range of hosts. Several representatives of this group are responsible for severe human diseases and for economic losses in agriculture and livestock. While this group encompasses over 30 genera, most of the available information has been derived from the vertebrate pathogenic genera *Leishmania*and *Trypanosoma.* Recent studies of the previously neglected groups of Kinetoplastea indicated that the actual diversity is much higher than previously thought. This article discusses the known segment of kinetoplastid diversity and how gene-directed Sanger sequencing and next-generation sequencing methods can help to deepen our knowledge of these interesting protists.

## Overview of kinetoplastid classification and diversity

**Fig. 1:**
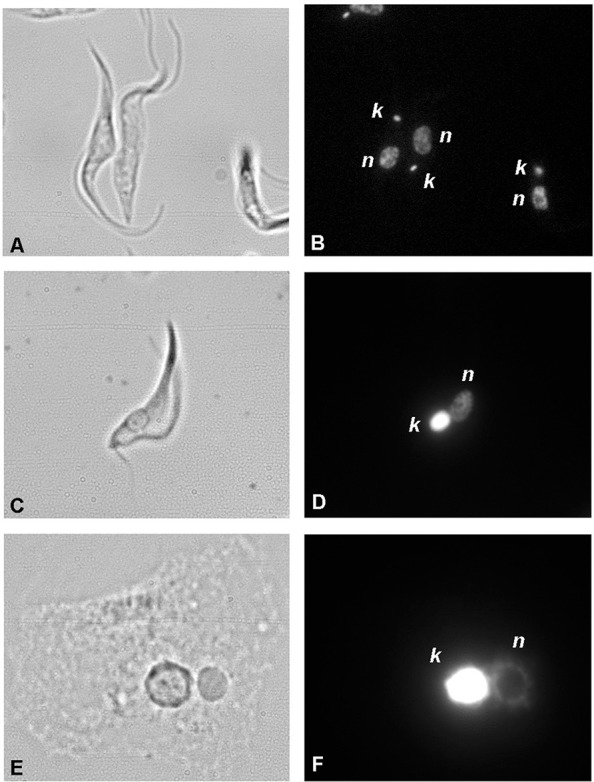
: images of the main patterns of kinetoplast DNA arrangement. Eukinetoplast of *Trypanosomabrucei* (A, B), pankinetoplast of *Trypanoplasma borreli* (C, D) and polykinetoplast (E, F) of *Perkinsela* sp. (A, C, E) bright field (B, D, F) DAPI staining. k: kDNA; n: nucleolus.

Kinetoplastid protists belonging to the phylum Euglenozoa (Cavalier-Smith 1981) are characterised by the presence of a kinetoplast, which is the apomorphy for the group and which is easily identifiable as a large mass of mitochondrial DNA (kDNA) (Vickerman & Preston 1976, Adl et al. 2012). The distribution of kDNA within the mitochondrion has three patterns: compacted and lying close to the flagellar pocket (termed eukinetoplast), dispersed throughout the mitochondrial lumen in several identical clusters (termed polykinetoplast), or unevenly dispersed as a diffuse mass (termed pankinetoplast) (Fig. 1) (Lukeš et al. 2002, Moreira et al. 2004). The lifestyle (parasitic vs. free-living, monoxenous vs. dixenous, intracellular vs. extracellular, and others), disease manifestation, and morphological traits have historically been used to classify these organisms (Lukeš et al. 2014, Votýpka et al. 2015a).

**Fig. 2:**
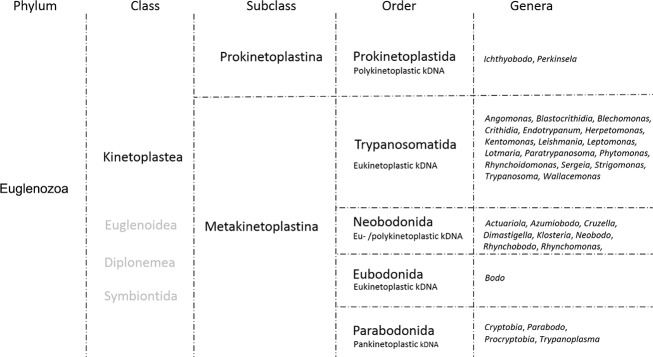
: updated taxonomy of kinetoplastids. The phylum Euglenozoa (Cavalier-Smith 1981) encompasses five classes, among which the class Kinetoplastea is subdivided into two subclasses. The bulk of the diversity described is within the Metakinetoplastina that is further subdivided into four orders. The order Kinetoplastida encompasses representatives responsible for human diseases and contains the largest number of described genera and species. This organogram compiles taxonomic data from Moreira et al. (2004) and Adl et al. (2012). It should be pointed out that the National Center for Biotechnology Information database still uses the formerly accepted classification, i.e., order Kinetoplastida encompassing three families: Bodonidae, Ichthyobodonidae and Trypanosomatidae.

Recently, 18S (small subunit) rRNA-based phylogenetic analyses have led to extensive changes in the classification of kinetoplastid flagellates. The class Kinetoplastea, hierarchically equivalent to the formerly accepted order Kinetoplastida, is now divided into two subclasses: Prokinetoplastina and Metakinetoplastina (Moreira et al. 2004, Adl et al. 2012). The latter brings together four orders, of which the Trypanosomatida contains the majority of catalogued species. Notably, the National Center for Biotechnology Information database still uses the former version of classification, i.e., order Kinetoplastida, encompassing the families Bodonidae, Ichthyobodonidae and Trypanosomatidae (Fig. 2).

The order Trypanosomatida encompasses parasitic species responsible for economic losses in agriculture and livestock and for severe human diseases. The order Trypanosomatida is composed of a single family, Trypanosomatidae, which covers a diverse group of strictly parasitic uniflagellated protists with either monoxenous or dixenous life cycles. Regarding the latter, Chagas disease, leishmaniases and African sleeping sickness are diseases caused by *Trypanosoma cruzi*,*Leishmania* spp. and two subspecies of *Trypanosoma brucei* (*T. b. rhodesiense* and *T. b. gambiense*), respectively, and these diseases affect millions of people worldwide (Vickerman 1994, Stuart et al. 2008). In addition to humans, a wide range of domestic and wild animals can be infected by *T. b. brucei*, *Trypanosoma congolense* and *Trypanosoma vivax*, which are responsible for a complex of animal trypanosomiases in Africa that are collectively called *nagana*. *T. b. evansi* causes a globally distributed disease called*surra* in domestic and wild animals found in Asia, Africa, South America, and Europe (Carnes et al. 2015), and several other species can occasionally cause atypical human trypanosomiases (Truc et al. 2013). Moreover, new clades of potentially pathogenic trypanosomes are emerging in phylogenetic trees, further expanding the landscape of African trypanosomes (Votýpka et al. 2015b). Interestingly, some *Trypanosoma* or *Leishmania*species are nonpathogenic to mammals and can infect hosts such as lizards, fish, snakes and frogs (Simpson 1986, Simpson et al. 2006, Viola et al. 2009, Zídková et al. 2012, Grybchuk-Ieremenko et al. 2014, Stoco et al. 2014, Ferreira et al. 2015). Moreover, several*Phytomonas* species can cause damage to economically important fruits and plants such as coffee, corn, coconut, oil palm, and cassava, although the phytopathology is not well established (Dollet 1984, Camargo 1999, Jaskowska et al. 2015).

**Fig. 3:**
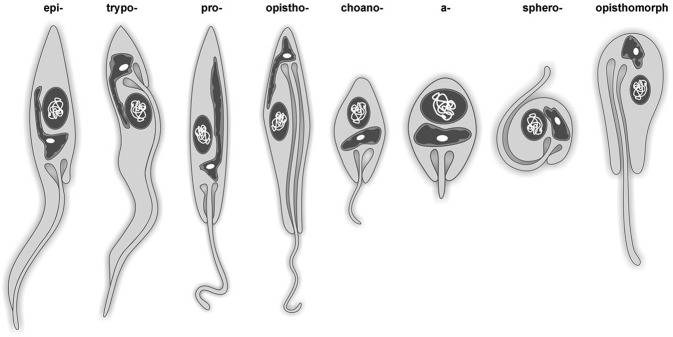
: schematic representation of the main morphological forms present in trypanosomatids. The main typical morphotypes observed are represented; the dash should be replaced by the word “mastigote”.

The most comprehensive and up-to-date catalogue of trypanosomatid genera and species was published 25 years ago and described known species, their synonymies, hosts, and distribution (Podlipaev 1990). Since then, substantial progress has been made in systematics and taxonomy primarily due to the introduction of molecular approaches. For a long time, trypanosomatid taxonomy was based solely on morphology and life cycles (Hoare & Wallace 1966, Vickerman 1976, McGhee & Cosgrove 1980), yet both parameters have a range of limitations, with morphology requiring the examiner to have a high level of proficiency (Fig. 3).

**Fig. 4:**
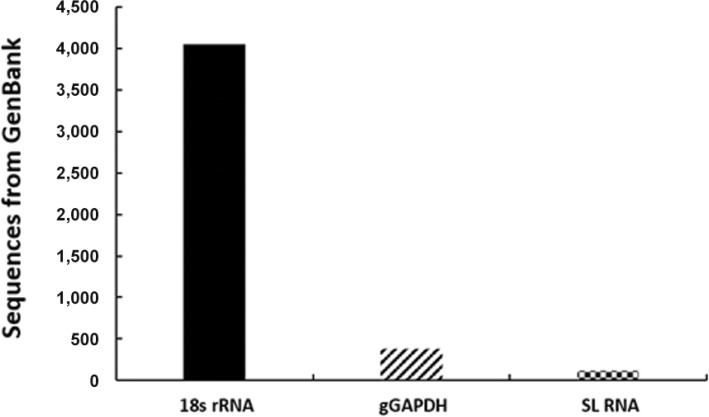
: comparison on the number of available kinetoplastid sequences from GenBank database. The most abundant sequences are: 18S (small subunit) rRNA gene, glycosomal glyceraldehyde phosphate dehydrogenase (gGAPDH) and spliced leader (SL) RNA. Mitochondrial cytochrome b, internal transcribed spacer one (ITS1) or two (ITS2) of rRNA, glucose 6 phosphate isomerase and the 70 kilodaltons heat shock protein also possess a considerable number of sequences, yet concentrated on *Leishmania*and *Trypanosoma*.

However, during the last decade, the traditional taxonomy has been integrated with DNA sequencing data. The 18S rRNA gene, glycosomal glyceraldehyde phosphate dehydrogenase (gGAPDH) and spliced leader (SL) RNA gene repeats are the most commonly used markers for molecular phylogenetic reconstructions of kinetoplastid flagellates (Maslov et al. 1996, Croan et al. 1997, Lukeš et al. 1997, Hollar et al. 1998, Yurchenko et al. 2000,Merzlyak et al. 2001, Hamilton et al. 2004, Teixeira et al. 2011, Borghesan et al. 2013) (Fig. 4). Using these molecular markers, species identification can be made by direct comparison with available DNA databases. However, if the match with the reference sequence is not full, the identification depends on accurate interpretation of molecular phylogenetic reconstructions and a rather arbitrary decision regarding whether the difference is intraspecific, interspecific or intergeneric. Commonly, the reference sequences are not correctly reassembled to updated taxonomic reclassifications, thus creating another challenging task of correctly comparing new isolates to previously described species.

In this sense, it is not surprising that our knowledge about the apparently extensive diversity of this group of protists remains fragmented. Moreover, a taxonomic bias towards vertebrate pathogenic species exists; this bias improves our knowledge of their nutritional requirements, therefore favouring their isolation and cultivation in vitro, leaving a vast segment of the free-living species diversity unexplored. Indeed, the order Trypanosomatida has more described genera and species than the sum of the other four orders (Fig. 2).

Insect and plant trypanosomatids, although not usually pathogenic to humans, have been widely used in basic research as model organisms to unveil aspects of cellular biology, biochemistry and genetics and in the search for antitrypanosomatid drugs (Hoare & Wallace 1966, Vickerman & Preston 1976, McGhee & Cosgrove 1980, Camargo 1999). Another possible explanation for the great expansion of the known trypanosomatid diversity not correlated with that of other groups of Kinetoplastea may be morphological uniformity and, hence, wide occurrence of cryptic species (Von der Heyden & Cavalier-Smith 2005). Exploring the diversity of the entire Kinetoplastea class is thus relevant for (i) filling the gaps in the tree of life, which would help to reconstruct more robust phylogenetic and evolutionary histories, (ii) comprehension of protistan synecology (i.e., the composition of their communities) and (iii) diversity inventory and conservation for future generations.

The primary aim of this article is to discuss achievements and potentials to screen kinetoplastid diversity directly within the hosts and in the environment using modern molecular approaches.

## Kinetoplastid diversity screen in the metagenomics era

Thus far, diversity and taxonomy studies have been based on polymerase chain reaction (PCR) amplification of molecular markers followed by DNA sequencing. This field is facing a dynamic and tremendous revolution. Over the past decade, the development of generations of sequencing technologies has resulted in an almost exponential increase in throughput and accuracy. Despite being relatively new, current sequencing techniques and associated bioinformatics analyses are now highly accurate and reasonably priced, with whole-genome sequencing of eukaryotes becoming a standard approach.

Complete genomic data of reference organisms are the best sources of information for diversity and phylogenetic studies. However, free-living protist genome projects encompass only a small fraction of completed and ongoing eukaryotic genome projects (Dawson & Fritz-Laylin 2009, del Campo et al. 2014), and the primary impediment to sequencing genomes is the scarcity of representative free-living protists in stable, axenic cultures (Dawson & Fritz-Laylin 2009). From 2,213 fully sequenced eukaryotic genomes, 59 belong to kinetoplastid protists, the majority of which pertain to the genera *Leishmania* (n = 24) and*Trypanosoma* (n = 16); these genera are over-represented due to their medical importance. The other genera with available genomic data are as follows: *Crithidia* (n = 3), *Leptomonas* (n = 2),*Trypanoplasma* (n = 1), *Strigomonas* (n = 4),*Angomonas* (n = 3), *Lotmaria* (n = 1),*Herpetomonas* (n = 1), *Endotrypanum* (n = 1),*Bodo* (n = 1) and *Phytomonas* (n = 3) (ncbi.nlm.nih.gov/genomes, sanger.ac.uk, tritrypdb*.*org). High-quality, well-annotated genomes are available for trypanosomatids. Additionally, molecular tools, such as gene knockouts, ectopic gene expression, RNAi and CRISPR, have been developed to improve genome annotation and to determine gene function and localisation (Dean et al. 2015). New bioinformatics tools for reanalysis of genome databases allow further identification of “partial” genes that can be categorised as C-terminal extensions, gene joining, tandemly repeated paralogs and wrong chromosomal assignments (Pawar et al. 2014).

The microeukaryotic diversity that resides in ecological niches such as animal microbiotas (for instance, insect gut or salivary glands), lakes, oceans and soil remains poorly understood (Foster et al. 2012, Weinstock 2013). Furthermore, any existing relationships among these species remain largely undiscovered. Due to the reduction in costs, labour intensity and time, new generation sequencing has the potential to reveal both the diversity and/or ecological and metabolic functions in virtually any environment. A recent salient example is the qualitative and quantitative new insights into this problem achieved by the Tara Oceans project, which not only massively extended the known eukaryotic diversity in the world oceans (de Vargas et al. 2015), but also explored a wealth of putative interactions among them (Lima-Mendez et al. 2015). However, because DNA sequencing from environmental samples generates a large amount of information, correctly and clearly formulated questions are of major importance.

The concept of DNA metabarcoding relies upon the identification of species present in environmental samples directly, without the need for microscopic observation or cultivation. This method is performed by direct extraction of DNA and PCR amplification of a selected gene (fragment) used to barcode the targeted group of eukaryotes (Pompanon et al. 2011, Epp et al. 2012, Taberlet et al. 2012, Aylagas et al. 2014, Pompanon & Samadi 2015). The metabarcoding approach aims to answer the following question: who is out there? In contrast, metagenomics aspires to functionally analyse the whole DNA present in a given sample from the perspective of the following question: how does the organismal assembly function? The two approaches have thus far been used by the research community somewhat indistinctly, although a distinction is advisable (Mendoza et al. 2015).

The utilisation of DNA sequences of short standardised gene fragments for quick and accurate determination of the species is called DNA barcoding. Because no consensus of a single marker able to distinguish and classify all the species on the planet exists, group-specific markers have been proposed (Pawlowski et al. 2012) (also see BOLD; boldsystems.org/). The regions of the mitochondrial gene encoding cytochrome *c* oxidase subunit 1 (CO1) and mitoribosomal RNAs are used for animals (Hebert et al. 2003), while two large subunits of the chloroplast RuBisCO and maturase K genes are used for plants, 16S rRNA for bacteria, internal transcribed spacer region 1 for fungi, and some other genes for less studied groups (Pawlowski et al. 2012). Although CO1 was shown to be insufficient for species delimitations for many microorganisms (Begerow et al. 2010, Pawlowski et al. 2012, Lebonah et al. 2014), it is applicable to a number of eukaryotic groups including trypanosomatids (Chantangsi et al. 2007,Nassonova et al. 2010, Stern et al. 2010, Kher et al. 2011, KA Morelli et al., unpublished observations). However, a consensual barcoding approach for kinetoplastids does not exist, although barcoding by means of 18S rRNA and gGAPDH is applied frequently.

The majority of the microeukaryotic diversity remains undiscovered primarily due to the methodological approaches used to assess it. While prokaryotic diversity studies are based mainly on 16S rRNA sequencing of their communities, for historical reasons, protistan diversity described without the establishment of axenic cultures and/or microscopic observation was considered incomplete and insufficient during the genomic era (Votýpka et al. 2015a). The identification of a kinetoplastid species has been traditionally based on its introduction into an axenic culture, with the culture-dependent approach considered critical for species validation. However, although establishment in culture is not feasible in many cases, the metabarcoding approach is not yet widely used even in studies of protistan diversity (Stoeck et al. 2005, Von der Heyden et al. 2005, Sauvadet et al. 2010, McCarthy et al. 2011, Bates et al. 2013, Glaser et al. 2014). Other hurdles include the low number of reference genomes in databases available for comparison and difficulties in establishing universally accepted markers (Sturm et al. 2008), as discussed above. In many cases, culture establishment is prevented by our limited knowledge of kinetoplastid metabolism and nutritional requirements, which is improving at a very slow pace even in well-studied groups (Škodová-Sveráková et al. 2015). Consequently, we are confined only to the cultivable fraction of protist diversity. Direct microscopic observation of environmental samples provides substantial morphological and ecological data related to eukaryotic communities in vivo*.* However, these data are hard to compare with the existing formally recognised species primarily due to high morphological variability (Dawson & Fritz-Laylin 2009, Votýpka et al. 2015a). Culture-independent approaches to assess diversity, such as single-cell sequencing methodology, which was recently successfully applied to protists (Kolísko et al. 2014), should help address these questions. Overall, the exploration of protistan diversity in general and kinetoplastid diversity in particular, appears significantly restrained by established and rather rigid traditional approaches.

## Genes used for molecular phylogeny of kinetoplastids

The SL RNA gene has been repeatedly used to explore trypanosomatid diversity using either parasites isolated in culture or direct insect gut contents, allowing many new trypanosomatid taxa to be described (Westenberger et al. 2004, Maslov et al. 2007, 2010, Yurchenko et al. 2008, 2009, Votýpka et al. 2010, 2012, 2013, 2014, Wilfert et al. 2011). This gene is absent from host genomes and from nonkinetoplastid microorganisms that could occur within such samples (Westenberger et al. 2004). The SL RNA gene consists of regions with different levels of variability (exon, intron, and intergenic spacer variability), which makes this gene suitable for both inter and intraspecific comparisons. Additionally, differences in the product amplification length among trypanosomatid species often allow the detection of mixed infections by standard agarose gel electrophoresis.

Species discrimination using the SL RNA gene is based on a 90% sequence similarity threshold (Westenberger et al. 2004). Although this criterion is arbitrary, it has withstood scrutiny and has provided a simple operational rule necessary for broad-scale studies. Hence, this criterion is an integral part of taxonomic studies of insect trypanosomatids (Kostygov et al. 2014).

Meanwhile, using the SL RNA gene in diversity studies has several disadvantages, particularly for PCR-based approaches. First, universal primers for this marker are not suitable for its amplification in some trypanosomatids (Podlipaev et al. 2004), making its use for metabarcoding analysis of the entire Kinetoplastea class questionable. Thus, SL RNA-based mapped diversity may be narrower than the actual diversity. Second, the very short conserved region of the gene (the exon and intron together are approximately 100 bp in length) does not provide sufficient data for deeper phylogenetic analysis. Third, different SL RNA gene classes varying in size and sequence have been described in a few species (Lamontagne & Papadopoulou 1999), yet this finding was not confirmed by whole-genome analyses (Berriman et al. 2005, Thomas et al. 2005). Fourth, the size differences of SL RNA gene repeats lead to competitive amplification favouring shorter PCR products. Hence, in the case of mixed infections, some species with longer repeats may remain undetected; this particular issue can be effectively addressed using new generation sequencing.

Due to these disadvantages, several research groups adopted a more habitual marker in diversity studies, the 18S rRNA gene, which can be amplified either from environmental samples or from cultured materials. The usage of different kinetoplastid-specific primers allows either the nearly complete gene or its most variable part to be obtained (Maslov et al. 1996, Kostygov & Frolov 2007,Votýpka et al. 2015b). Thus far, the 18S rRNA gene has been successfully used in diversity studies not only for insect trypanosomatids (Votýpka et al. 2010, 20,^2012^, Týč et al. 2013), but also for fish trypanosomes and trypanoplasms (Grybchuk-Ieremenko et al. 2014, Losev et al. 2015), as well as for flagellates from deep-sea samples (Sauvadet et al. 2010, Scheckenback et al. 2010, Pawlowski et al. 2011, Salani et al. 2012d, de Vargas et al. 2015). A few reports used the 18S rRNA gene to scrutinise lake sediments (van Hannen et al. 1999) and soil (Glaser et al. 2014). No generally accepted criterion of species discrimination exists based on this gene most likely due to its unpredictable variability in different groups of eukaryotes. For example, the observed multiple closely related haplotypes of this gene in trypanosomes parasitising fishes suggest that some intraspecific variability of this marker exists within the given group (Grybchuk-Ieremenko et al. 2014).

## Assessment of molecular diversity by metagenomic approaches

Comprehensive assessment of the molecular diversity of unicellular eukaryotes retrieved from deep-sea water has been the focus of several studies in the past 15 years. Although prokaryotic communities have been studied extensively, protists have been generally much less explored in aquatic environments, where they thrive even under conditions of high pressure, high toxic product concentrations and high and low temperatures. A study devoted to revealing microeukaryotic diversity in the abyssal sea floor of the Atlantic Ocean used general eukaryotic and kinetoplastid-specific primers to discover members of the genera*Ichthyobodo*, *Rhynchobodo* and*Neobodo* (Scheckenbach et al. 2010). In cultivation-independent studies of the South Atlantic, Mediterranean and other sites, kinetoplastid-specific 18S rRNA primers were used to detect *Neobodo designis*, *Rhynchobodo* sp. and*Ichthyobodo*. Notably, a particular percentage of identical clones is shared among even geographically distant regions, suggesting global distribution (Von der Heyden & Cavalier-Smith 2005, Salani et al. 2012). Protist community surveys from deep-sea waters from hydrothermal vents in the Pacific Ocean using general 18S rRNA primers revealed the presence of *Bodo* sp. and *Bodo saliens* (Brown & Wolfe 2006, Sauvadet et al. 2010). In other hydrothermal areas in the Mid-Atlantic Ridge and the eastern Pacific Ocean, kinetoplastids such as *Ichthyobodo necator*, *Procryptobia sorokini*, *Rhynchomonas nasuta*,*Bodosaltan*s and *B. saliens* were also abundant (Atkins et al. 2000, López-García et al. 2003). Although these data reveal the ubiquitous distribution of kinetoplastids and their exciting plasticity, which allows them to adapt to extreme environments, no cultured representatives from these environments are available. In spite of these advances, deep-sea kinetoplastid sequences have disproportionally low representation in public databases (Salani et al. 2012). An extensive 18S rRNA metabarcoding study of the sunlit zone of the world oceans by the Tara Oceans initiative revealed a surprisingly highly abundant presence of diplonemids (Lukeš et al. 2015) and a much less conspicuous presence of kinetoplastids (de Vargas et al. 2015). In another 18S rRNA-based survey targeting aquatic microeukaryotes in The Netherlands, sequences related to parasitic trypanosomatids have been described (van Hannen et al. 1999). However, their re-analysis against recently available sequences revealed their high identity with *N. designis* (KA Morelli, unpublished observations).

A cultivation-independent survey of kinetoplastid diversity in soil employed 18S rRNA primers and revealed an abundance of sequences related to the neobodonid clade, followed by parabodonids and eubodonids (Glaser et al. 2014). While approximately 30% of the obtained sequences have low similarity to databases, whether these sequences are derived from unknown taxa, the so-called rare biosphere, or represent methodological “noise” remains to be established (Glaser et al. 2014). In a study that aimed to investigate the role of free-living protists in contaminated food,*Bodo* sp. and *Parabodo caudatus* were frequently detected, along with related sequences with low BLAST scores (Vaerewijck et al. 2008).

Collectively, these data emphasise the need for more comprehensive studies targeting free-living kinetoplastids, the diversity of which remains fractionated, underestimated and, consequently, poorly taxonomically and phylogenetically studied. As a result of the increasing application of 18S rRNA gene-based approaches, new protistan phylotypes are constantly being revealed (López-García et al. 2001, Taib et al. 2013), improving our knowledge of the diversity, distribution and function of eukaryotic microorganisms.

## Museums and institutional collections as a basis for diversity screening

In comparison to macroscopic eukaryotes, protist collections are generally unknown to the public certainly because they concern microscopic organisms that are not spectacular or emblematic. These collections are often accumulated in dusty boxes of slides stored on shelves in an obscure corner. However, for protistologists, such collections are gold mines primarily because they contain type material (hapantotypes) deposited since the end of the XIX century by generations of scientists (Votýpka et al. 2015a).

With the beginning of the molecular era in the 1990s, natural history collections evolved to meet the challenges of the current and future interdisciplinary studies. Many institutions developed new collections and information databases (DNA, tissues, cultures, cryobanks, photographs, ethanol-fixed specimens, publication collections, and geographical and ecological information databases), which are of first-rate importance, offering opportunities to conduct integrative studies, including temporal and spatial surveys (Suarez & Tsutsui 2004).

With the worldwide awareness of the dramatic erosion of both macro and microorganismal diversity, the necessity of its inventory and preservation is now a priority. Many museums and academic institutions are engaged in large surveys in diversity host spots [see for example laplaneterevisitee.org/en/ (Bouchet et al. 2008)]. In addition to traditional taxonomy, DNA barcoding approaches are used to describe diversity. Furthermore, recent works have demonstrated the possibility of extracting relevant genetic information from ancient archived specimens such as archaeological remains (Frías et al. 2013), formalin-fixed tissues (Gilbert et al. 2007), and fixed and stained smears (Hayes et al. 2014). For a long time, such material was considered useless for molecular analyses due to DNA degradation. Studies on ancient human remains have changed the widely accepted theory of the origin of Chagas disease. Approximately 9,000-year-old pre-Colombian mummies were shown to be PCR-positive for *T. cruzi*, indicating that Chagas disease is at least as old as human presence in the Americas (Aufderheide et al. 2004). Another example derived from the museum collections is the rapid extinction of endemic rats on Christmas Island around the year 1900 due to *Trypanosoma lewisi*introduced by black rats and their fleas (Wyatt et al. 2008).

The possibility of extracting DNA suitable for amplification from fixed and stained blood smears and other difficult samples opens new avenues for the molecular characterisation of kinetoplastid type specimens deposited in collections. Their potential use in studying kinetoplastid diversity can be illustrated by the recent work on trypanosomes of marine fishes from South Africa and their leech vectors (Hayes et al. 2014).

## Trends in metabarcoding of kinetoplastids

Direct sequencing of the environmental DNA, either total or focused on barcoding markers, has been the basis for “blind” diversity screens. After the early studies of diversity through direct DNA sequencing, the overall ratio of cultivable microorganisms has been generally accepted not to exceed 1% of the total diversity on earth (Pace 1997). For protists in particular, less than 10% of the sequences revealed by cultivation-independent molecular surveys were previously known (Šlapeta et al. 2005, Medinger et al. 2010). These approaches revealed not only putatively novel species, but also new kingdoms (Dawson & Pace 2002, Berney et al. 2004, Cavalier-Smith 2004). However, these data are problematic because nothing beyond the molecular signature is known, such as morphological and/or biochemical characteristics of the new organisms, their ecological roles, or in situ abundance. Hence, we can only speculate whether these distinct molecular signatures represent existing unknown microbes or are only methodological artefacts.

Although taxonomic information of an unknown microorganism through DNA sequencing is interesting *per se*, ideally, this information must be combined with morphological, biochemical and ecological data (Votýpka et al. 2015a). For example, in the order Neobodonida, an undescribed sequence indicated the existence of a novel clade that appeared to consist of free-living organisms from aquatic and terrestrial habitats (López-García et al. 2003, Von der Heyden & Cavalier-Smith 2005). However, no cultured representatives of this clade were available. Later, a diversity survey using combined molecular and culturing approaches succeeded in isolating and culturing an organism that branched within that undescribed neobodonid clade according to its phylogenetic position (Stoeck et al. 2005).

Another issue to consider while screening environmental sequences is whether the infrequent sequences are indeed members of a highly diverse microbial “rare biosphere” or only represent sequencing artefacts. To address this question, tintinnid ciliates, a species-rich group that can be easily distinguished morphologically, were surveyed to assess the accuracy of 18S rRNA pyrosequencing of Mediterranean samples with different patterns of tintinnid diversity. The inferred number of typing units outnumbered tintinnid cells in the samples, which was found to be primarily dependent on the data treatment, suggesting that many undescribed environmental sequences might indeed be artefacts (Bachy et al. 2013).

The intention of this review is to critically evaluate the usefulness of methodological advances for studies of kinetoplastid diversity. The scarcity of protist environmental data is a large obstacle for the perception of true eukaryotic diversity. An analysis of the SILVA SSU database of the eukaryotic phyla (Pruesse et al. 2007) showed that less than 5% of the 18S rRNA sequences originated from protists (Pace 2009). A recent re-evaluation of environmental studies revealed that protists that were previously overlooked constitute the bulk of extant eukaryotic diversity (Pawlowski et al. 2011).

Metabarcoding has become a fundamental approach for diversity assessment in recent years. The possibility of revealing previously unknown microorganisms through metabarcoding and the potential of unveiling their physiology and ecology through metagenomics pose great opportunities and challenges to protistologists.
